# Evaluation of dentin thickness preservation and the efficiency of instrumentation between traditional and guided endodontic access in mandibular central incisors

**DOI:** 10.1016/j.jobcr.2025.04.011

**Published:** 2025-05-08

**Authors:** Pooja R. Kesharani, Shalini D. Aggarwal, Nishtha K. Patel, Jhanvi Patel, Ankita Bansal, Naman Patel

**Affiliations:** a Dept. of Conservative Dentistry and Endodontics, College of Dental Sciences and Research Centre, Manipur, Ahmedabad, 380058, India; b Dept. of Conservative Dentistry and Endodontics, Dr. D.Y. Patil Dental College and Hospital, Dr. D.Y. Patil Vidyapeeth, Pimpri, Pune, Maharashtra, 411018, India; c Dept. of Public Health Dentistry, College of Dental Sciences and Research Centre, Manipur, Ahmedabad, 380058, India; d Dept. of Oral Medicine & Radiology, College of Dental Sciences and Research Centre, Manipur, Ahmedabad, 380058, India

**Keywords:** CBCT, Centering ability, Endodontic access cavity, Guided endodontic access cavity, Pericervical dentin, Remaining dentin thickness

## Abstract

**Introduction:**

Tooth substance loss during endodontic treatment is a major concern, especially in mandibular incisors due to their minimal tooth volume. Template-guided access cavities help preserve dentin and improve instrument centering. This in vitro study compares remaining dentin thickness (RDT) and centering ability of rotary instruments using both conventional and template-guided approaches in mandibular incisors.

**Objective:**

Comparative in vitro CBCT study on remaining dentin thickness and centering ability of rotary instrumentation in mandibular incisors using conventional vs. template-guided access cavity preparation.

**Methodology:**

Pre-treatment CBCT scans were taken of 80 mandibular incisors, to evaluate the existing dentin thickness and these were then divided into 2 groups of 40 teeth each. Conventional endodontic access cavities were made in Group −1, and guided access openings were done in Group – 2. Post-operative CBCT scans were taken to measure the RDT canal centering ability of each approach.

The data was examined using a one-way analysis of variance, followed by Tukey's post-hoc test for multiple pairwise comparisons, with a significance level set at p < 0.05.

**Results:**

The mean RDT was significantly higher in the group where a template-guided access opening was done. The statistical difference for RDT amongst both the experimental groups was highly significant at the Cemento-Enamel Junction and 9 mm from the root apex. Statistically significant results were obtained 6 mm level and insignificant result was obtained at 3 mm level from root apex. No significant differences in the centering ability ratio were observed between the Traditional Endodontic Cavity (TEC) and Guided Endodontic Cavity (GEC) at any level.

**Conclusion:**

Pericervical dentin was preserved more in guided access cavity preparation. The design of the access cavity preparation did not impact the centering ratio of the instruments used for shaping the root canals.

## Graphical abstract

**Figure:**
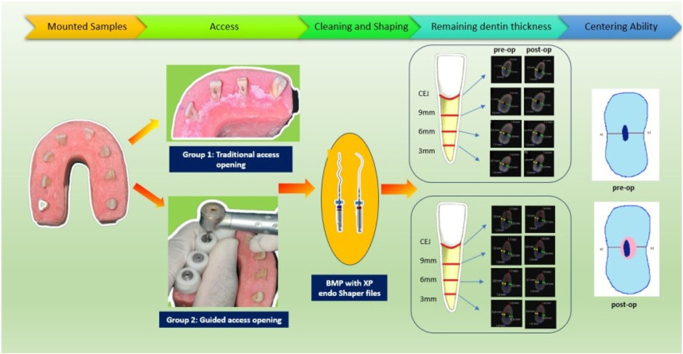

## 1 Introduction

Creating an endodontic access cavity is a vital step in root canal treatment. Successful treatment relies on its ability to facilitate thorough instrumentation, effective disinfection, and a three-dimensional filling of the canal system.^1^ In recent years, there has been a notable shift toward more conservative approaches in accessing canal systems. This shift has been introduced new terminology for access cavity preparation techniques. Terms such as conservative, ultra-conservative, truss access, Ninja access, and guided access represent approaches that have gained prominence in supporting successful root canal treatment.^2^

Conservative access cavities emphasizes dentin preservation and the integration between endodontic and restorative treatments. It prioritizes removing restorative material over tooth structure, enamel over dentin, and occlusal tooth structure over cervical dentin.^2^ This technique deviates from traditional practices by downplaying the necessity for straight-line access and complete unroofing of the pulp chamber, instead focusing on conserving the critical pericervical dentin.^3^

Guided access cavity preparation takes advantage of advanced technologies such as Cone Beam Computed Tomography (CBCT), CAD-CAM, and surface scans of the tooth. These tools allow for the creation of a three-dimensional static guide (static-guided navigation) or the ability to track a surgical instrument in real-time (dynamic navigation) to visualize its position while creating a precise drill path. This guided approach enables practitioners to access the apical portion of the tooth with precision.^4^ The concept of 3D static-guided endodontic access was first introduced by Byun et al. (2015)^5^ and Zubizarreta Macho et al. (2015).^6^ Since then, numerous studies have been conducted to explore the clinical implications and benefits of this method.

The advent of modern rotary file systems promises simplified canal preparation, reduced procedural errors, and shorter preparation times.The unique properties of NiTi alloy enable it to navigate canals in a continuous rotating motion without experiencing permanent plastic deformation, resulting in more centered preparations. This helps maintain the original anatomy of the root canal structure.^7^

Previously, various methods were used to assess canal transportation, remaining dentin thickness, and centering ability, including radiographs, serial sectioning, photographic analysis, scanning electron microscopy, and computer-based techniques. However, these techniques were often invasive, and repositioning specimens accurately after instrumentation posed significant challenges. Additionally, traditional radiographs only provided two-dimensional (2D) images of three-dimensional (3D) structures.^8^

A more contemporary method employs non-invasive technology to evaluate canals before and after instrumentation. CBCT uses a cone-shaped X-ray beam and an area detector to acquire a cylindrical volume of data in a single scan. CBCT offers numerous benefits, such as producing highly accurate cross-sectional and 3D images with excellent resolution. It provides fully quantifiable data and ensures consistent and repeatable outcomes.^9^

There is limited literature available that compares guided endodontic access preparation with traditional methods, particularly in combination with mechanical root canal preparation using the XP Endo file system. Therefore, this study aims to compare and evaluate the remaining dentin thickness and centering ability in lower central incisors, utilizing both traditional and guided access preparation methods.

The null hypothesis is there is no difference in the remaining dentin thickness and centering ability in lower central incisors between traditional and guided access preparations.

## 2 Materials and methodology

Ethical approval was obtained prior to the study and number is CDSRC/IES/2024/35. A total sample size of 40 was selected, with a significance level of 5 % and a two-sided test used to calculate the sample size. Forty freshly extracted mandibular incisors were chosen for the study. The samples were cleaned, disinfected, and inspected under an operative microscope (Labomed Inc., USA) at 1.6 × magnification to assess their eligibility. They were then stored in saline for future use. Teeth with a single root and an oval canal, 3 mm short of the apex (determined via bucco-lingual and mesio-distal radiographic projections), and mature apices were included. Exclusion criteria consisted of deciduous teeth, teeth with multiple roots, cracks, caries, calcifications, previous restorations, fractures, internal resorption, or curvature in the coronal and middle thirds.

To ensure consistency and minimize bias, all the procedures were performed by a single operator. To simulate periodontal tissue, the samples were coated with modeling wax, and positioned parallel to one another, with the buccal surface facing outward to mimic the dental arch. They were then mounted in cold-cure acrylic resin to replicate alveolar bone.

The mounted assembly was subjected to pre-operative CBCT analysis to obtain volumetric and linear measurements. These measurements were taken at the cementodentinal junction (CDJ), as well as at 3 mm, 6 mm, and 9 mm from the apex, in all directions (mesial, distal, buccal, and lingual) using Ez-3Di software (version 5.0.0.2), from Vatech Company, South Korea.

The samples were then divided into two groups: Group 1 – Samples with traditional access opening, and Group 2 – Samples with guided access opening.

Group 1: In this group, access openings were performed using a size 2 round bur and an Endo-Z bur (Dentsply Maillefer, Ballaigues, Switzerland) under an operating microscope.
Group 2: Data from the pre-operative CBCT scans, stored in DICOM format, was converted to STL files, enabling the creation of a 3D template. Access openings were made using an EndoGuide bur no. 3 (with a tip diameter of 1.1 mm) in combination with the 3D template, also under an operating microscope.

A 15 K file was inserted into each sample to establish the working length, glide path, and patency (until the tip was visible at the apical foramen) using the operating microscope. Chemomechanical preparation was conducted using three cycles of the XP-Endo Shaper instrument at 800 rpm with 1 Ncm torque, operating in an in-and-out motion with a 3–4 mm amplitude up to the working length for 10 s. Irrigation was performed simultaneously with 2.5 % sodium hypochlorite for 30 s. The final irrigation sequence included 17 % EDTA for 30 s, followed by 2.5 % NaOCl for 30 s, and 10 % sodium thiosulfate for 1 min, with ultrasonic agitation using ultrasonic tips.

**Fig. 1:**
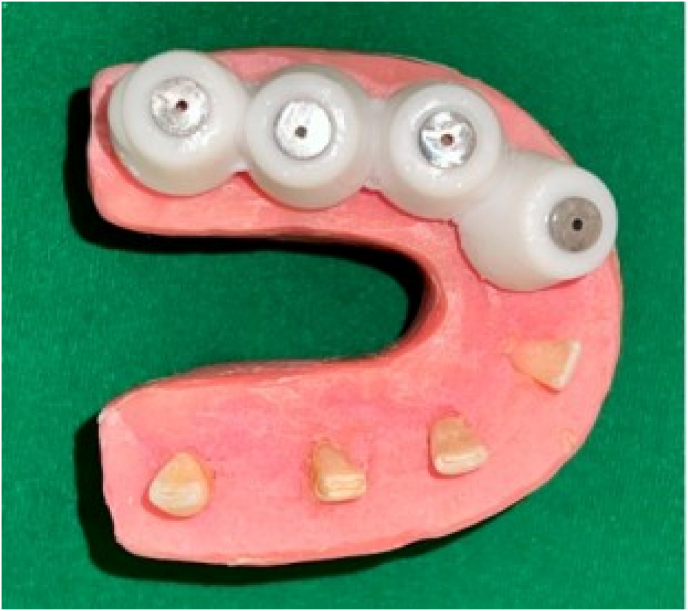
Mounted samples and 3D printed guide.

**Fig. 2:**
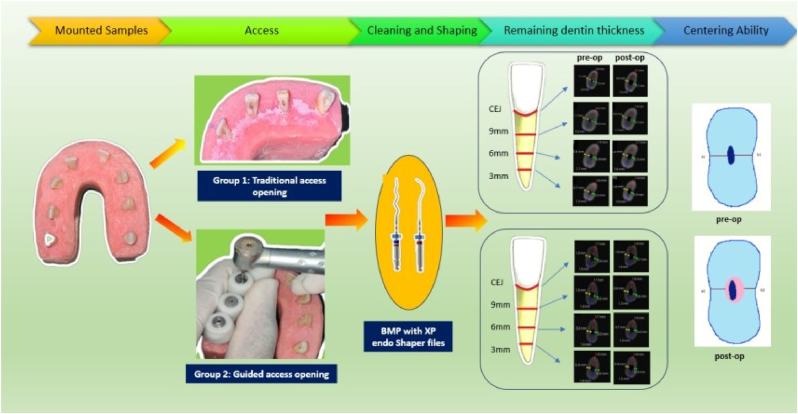
Graphical representation of materials and method.

All samples were then remounted into wax blocks, and post-operative CBCT scans were performed to assess the remaining dentin thickness and centering ability [Fig. 1, Fig. 2].

Measurement of remaining dentin thickness:

The RDT was calculated by subtracting the width of the uninstrumented canal from the width of the instrumented canal, using formulas (a1−a2) − (b1−b2). Here, a1 represents the distance from the mesial wall of the unprepared canal to the mesial wall of the root, and b1 is the distance from the distal wall of the unprepared canal to the distal wall of the root. Similarly, a2 denotes the distance from the mesial wall of the prepared canal to the mesial wall of the root, and b2 is the distance from the distal wall of the prepared canal to the distal wall of the root.

Meaurement of centering ability:

Centering ability was determined by the formula:

(a1−a2) / (b1−b2) or (b1−b2) / (a1−a2)

If the numbers are not equal, the lower figure is considered the numerator, and a result of “1” indicates perfect centering.

## 3 Result

**Fig. 3:**
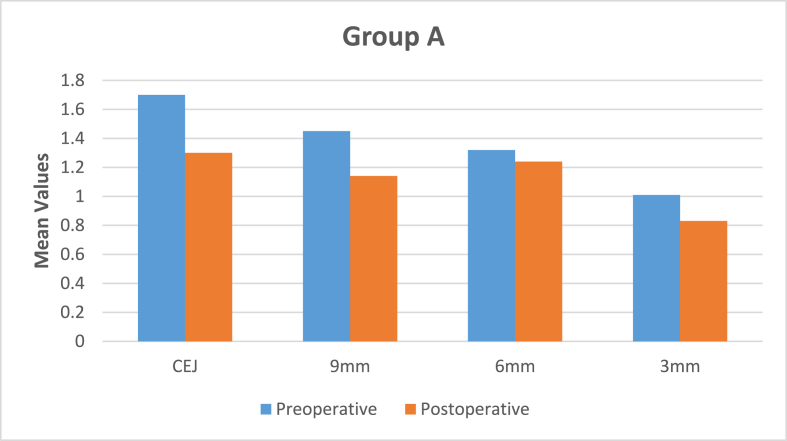
Comparison of pre-operative and post-operative RDT at all levels in Group A.

**Fig. 4:**
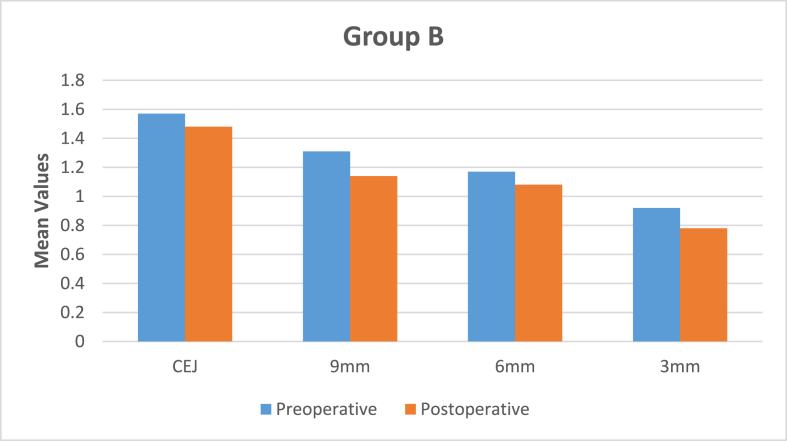
Comparison of pre-operative and post-operative RDT at all levels in Group B.

**Fig. 5:**
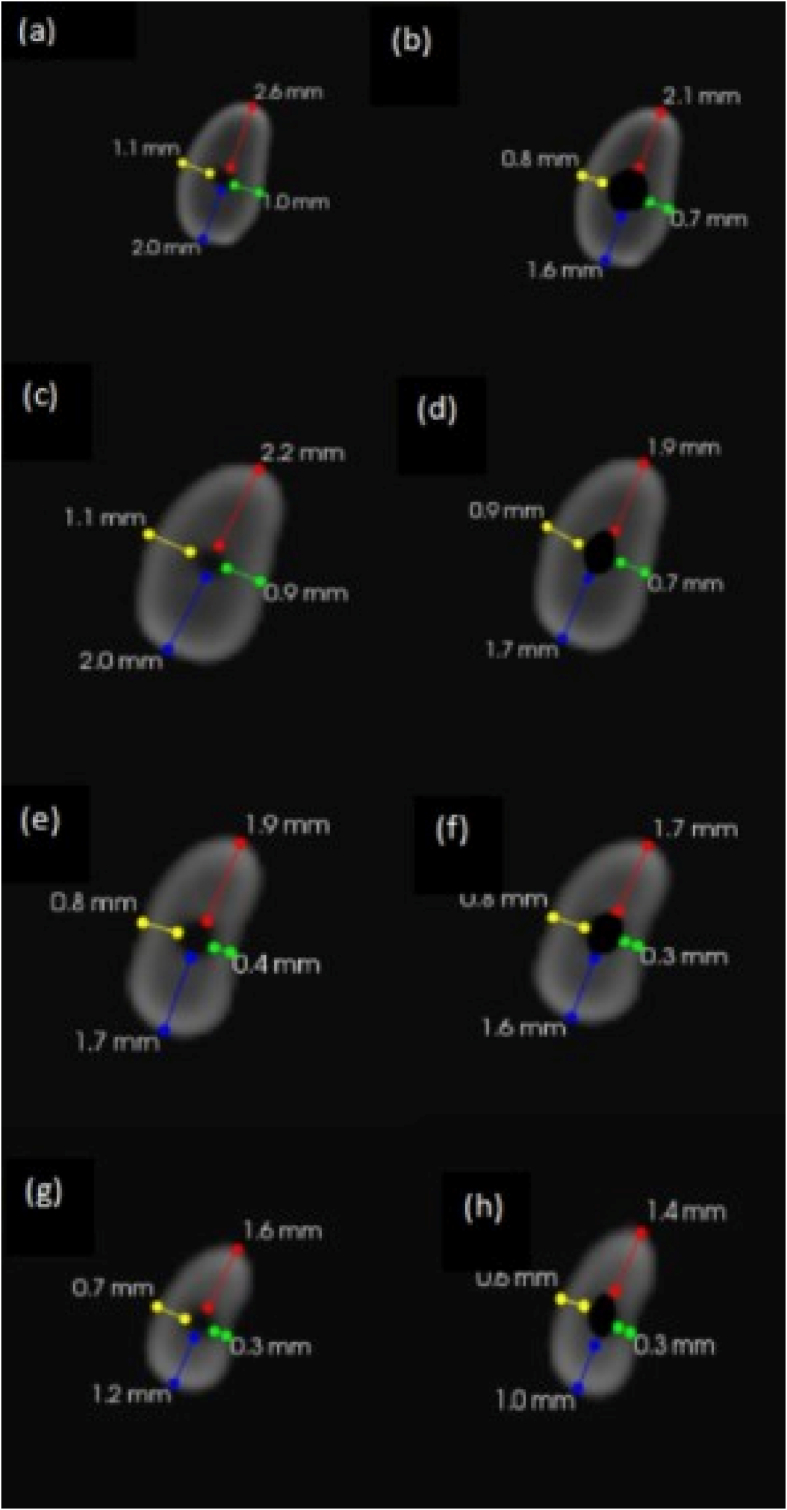
Pre-operative CBCT images for Group 1 (a) at DEJ (c) at 9 mm (e) at 6 mm and (g) at 3 mm. Post-operative CBCT images (b) at DEJ (d) at 9 mm (f) at 6 mm and (h) at 3 mm.

**Fig. 6:**
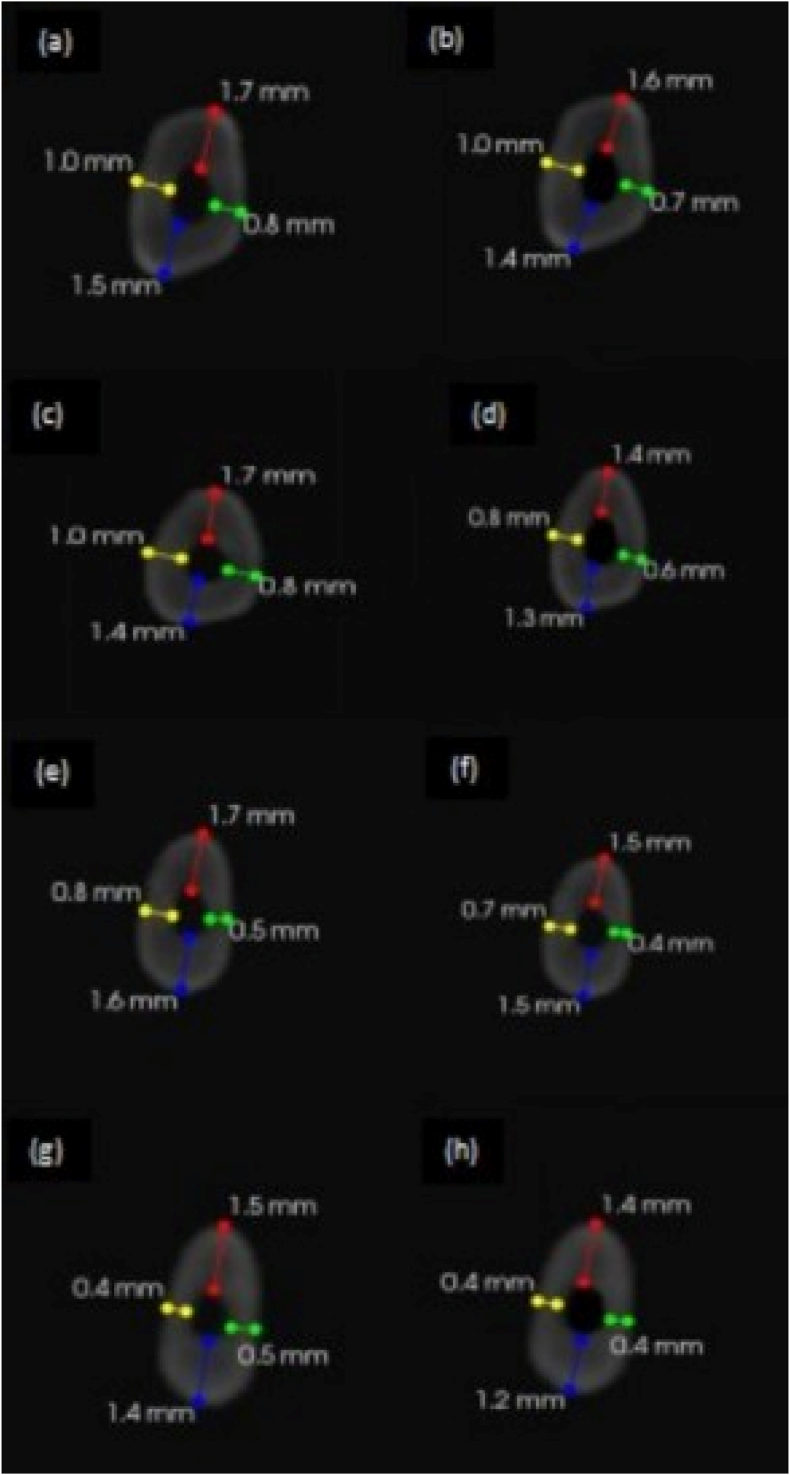
Pre-operative CBCT images for Group 2 (a) at DEJ (c) at 9 mm (e) at 6 mm and (g) at 3 mm. Post-operative CBCT images (b) at DEJ (d) at 9 mm (f) at 6 mm and (h) at 3 mm.

**Fig. 7:**
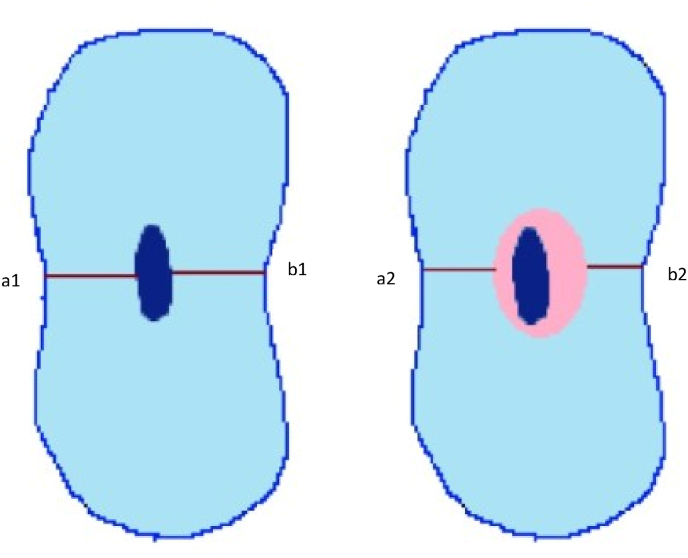
Schematic diagram of measurement of canal centering ability.

The data was entered and analyzed using the Statistical Package for Social Sciences (SPSS) for Windows, Version 28.0. (Armonk, NY: IBM Corp) Confidence intervals were set at 95 %, and a p-value ≤ of 0.05 was considered statistically significant. Paired *t*-test was applied to compare remaining dentin thickness (RDT) at CEJ, 3 mm and 9 mm for pre-operative and post-operative in Group A and Group B. Unpaired *t*-test was applied to compare Group A and Group B. There was high degree of statistical significance in pre-instrumented and post-instrumented RDT between both groups at CEJ and 9 mm levels on all the sides of the canal [Table 1, Table 4]. Statistically significant results were obtained in RDT between both groups at 6 mm level [Table 3] while there was no statistical significant difference in RDT between both groups at 3 mm from apex [Table 2] [Fig. 2, Fig. 3, Fig. 4, Fig. 5]. There was no statistically significant difference in canal centering ability at all levels of instrumented canal between both groups derived by unpaired *t*-test [Table 5] [Fig. 6, Fig. 7].

**Table 1:** Mean difference of remaining dentin thickness at cemento-enamel junction between two experimental groups and its level of significance.

	Group	n	Mean	SD	Mean difference	P
Buccal	A	20	0.54	0.37	0.44	0.0001∗∗∗
	B	20	0.1	0.25		
Lingual	A	20	0.43	0.34	0.35	0.00001∗∗∗
	B	20	0.08	0.30		
Mesial	A	20	0.34	1.83	0.25	0.00001∗∗∗
	B	20	0.09	0.34		
Distal	A	20	0.29	0.309	0.22	0.00001∗∗∗
	B	20	0.07	0.327		

p < 0.05: Statistically significant, ∗∗∗ highly significant.

**Table 2:** Mean difference of remaining dentin thickness at 3 mm between two experimental groups and its level of significance.

	Group	N	Mean	SD	Mean difference	P
Buccal	A	20	0.29	0.319	0.11	0.00∗
	B	20	0.18	0.173		
Lingual	A	20	0.24	0.287	0.08	0.02∗
	B	20	0.16	0.192		
Mesial	A	20	0.11	0.116	−0.003	0.00∗
	B	20	0.14	0.119		
Distal	A	20	0.11	0.109	0.002	0.00∗
	B	20	0.08	0.107		

p < 0.05 ∗: Statistically significant, ∗∗∗ highly significant.

**Table 3:** Mean difference of remaining dentin thickness at 6 mm between two experimental groups and its level of significance.

	Group	N	Mean	SD	Mean difference	P
Buccal	A	20	0.15	0.193	0.04	0.00∗
	B	20	0.11	1.59		
Lingual	A	20	0.09	0.261	−0.05	0.22
	B	20	0.14	0.17		
Mesial	A	20	0.06	0.141	0.02	0.27
	B	20	0.04	0.211		
Distal	A	20	0.04	0.168	−0.04	0.51
	B	20	0.08	0.251		

p < 0.05 ∗: Statistically significant, ∗∗∗ highly significant.

**Table 4:** Mean difference of remaining dentin thickness at 9 mm between two experimental groups and its level of significance.

	Group	N	Mean	SD	Mean difference	P
Buccal	A	20	0.34	0.187	0.11	0.00001∗∗∗
	B	20	0.23	0.215		
Lingual	A	20	0.30	0.209	0.11	0.00001∗∗∗
	B	20	0.19	0.276		
Mesial	A	20	0.30	0.123	0.17	0.00001∗∗∗
	B	20	0.13	0.162		
Distal	A	20	0.30	0.149	0.16	0.00001∗∗∗
	B	20	0.14	0.179		

p < 0.05: Statistically significant, ∗∗∗ highly significant.

**Table 5:** Mean and standard deviation values of centering ratio.

Level	Direction	Group A		Group B		p-value
		Mean	SD	Mean	SD	
At CEJ	D1 (B-L)	0.81	0.17	0.95	0.48	0.25
	D2 (M-D)	0.65	0.25	0.6	0.55	0.70
At 3 mm	D1 (B-L)	0.77	0.47	0.87	0.58	0.55
	D2 (M-D)	0.62	0.48	0.65	0.45	0.84
At 6 mm	D1 (B-L)	0.77	0.24	0.86	0.35	0.36
	D2 (M-D)	0.63	0.41	0.64	0.32	0.89
At 9 mm	D1 (B-L)	0.66	0.21	0.74	0.26	0.31
	D2 (M-D)	0.7	0.27	0.65	0.36	0.62

p < 0.05: Statistically significant, ∗∗∗ highly significant.

## 4 Discussion

Root canal therapy aims to treat an infected pulp, eliminate infection, and protect the tooth from future microbial invasion. One of the most pivotal steps in ensuring successful endodontic treatment is the precise preparation of the access cavity. The main objective of this step is to locate the root canal entrances, enabling efficient biomechanical preparation and obturation of the root canal system.^10^

The traditional "extension for prevention" approach aids treatment by improving visibility and access but often requires removing significant amounts of dentin in the cervical area, which can weaken the tooth structurally. In contrast, minimally invasive dentistry (MIE) has brought forward the concept of conservative endodontic access cavities. This modern approach prioritizes the preservation of healthy dentin, avoids fully removing the pulp chamber roof, and prevents excessive widening of canal orifices.^11^^,^^12^ It also stresses the importance of maintaining the integrity of the pericervical dentin, which is vital for the tooth's structural strength. MIE aims to preserve the healthy coronal, cervical, and radicular tooth structure.^12^

The trend toward creating smaller access cavities has been driven by advancements in magnification with enhanced illumination, the development of more flexible instruments, and improved imaging technologies like CBCT.^13^

The conservative endodontic cavity (CEC) concept, proposed by Clark and Khademi, focuses on preserving the pulp chamber roof and pericervical dentin. To achieve this, traditional round and GG burs should be avoided, as they are not self-centering and may cause gouging, which can complicate canal negotiation.^14^ Additionally, they remove excessive amounts of pericervical dentin and pulp chamber soffit, undermining a minimally invasive approach. Instead, EndoGuide burs, also known as CK burs, have been recommended by Dr. Clark and Khademi for their precision in magnification-driven endodontics.^15^

As highlighted by Lenchner NH et al., EndoGuide burs are ideal for use in endodontics that rely on magnification. In this study, a dental operating microscope (DOM) was utilized to enhance visibility during minimal cavity preparation. This allowed for the location of root canal orifices that are not in a straight line, detection of calcifications and obliterations, and reduced the risk of procedural errors such as gouging and strip perforation. Ultimately, the approach aimed to preserve more pericervical dentin, contributing to better long-term outcomes.^16^

In the present study, for standardization of methodology, the whole work was done by a single operator. CBCT imaging is a non-invasive tool used to superimpose pre- and post-instrumentation images to evaluate the geometry of root canals and assess the effectiveness of shaping procedures. Measurements can be taken at various levels of the root canal to determine centering ability and remaining dentin thickness (RDT).

In this study, four levels were selected: Cemento-enamel junction (CEJ) and 3, 6 and 9 mm from apex as minimum root length in mandibular central incisors is 12 mm. These levels correspond to the apical, middle, and coronal thirds of the root canal as well as CEJ area often prone to curvature and at higher risk for iatrogenic damage. The results revealed that Guided access cavities were able to preserve more dentine than Traditional access cavities, especially at CEJ and in the coronal third (9 mm from apex) of the canal. This result is in agreement with previous studies, which indicated that minimally invasive methods for endodontic access cavity preparation were associated with less dentine removal (Connert et al., 2019; Loureiro et al., 2020; Rover et al., 2017).

Statistically significant differences in RDT were observed between both groups at the 6 mm level and no significant difference was found at 3 mm from the apex. This may be due to the absence of straight-line access, which further hinders apical instrument control and can lead to complications in guided access cavities. This finding aligns with the study by Alovisi et al., which concluded that conventional endodontic access better preserves the original canal anatomy, particularly in the apical third, compared to guided endodontic access.

The centering ratio measures how well an instrument remains centered within the canal, which helps in evaluating the benefits and limitations of particular instrument designs.

Two crucial factors influencing both RDT and centering ability are the material composition of the instruments and their design characteristics, such as cross-section, taper, and tip shape.^17^ In this study, the shaping outcomes of the XP Endo Shaper and Finisher file system were analyzed. The XP Endo Shaper is made with MaxWire technology, which allows it to undergo phase transformation at body temperature. This enables the instrument to assume a snake-like shape that can expand and contract to adapt to the canal's natural morphology.^18^

However, one limitation of this study is that it was conducted in vitro, where replicating clinical scenarios can be challenging. It should also be considered that not all roots are cylindrical and centering in the canal might not always be desirable. Keeping in mind these variables further validation through clinical studies is necessary to confirm these results.

## 5 Conclusion

Pericervical dentin was preserved more in guided access cavity preparation. The design of the access cavity preparation did not impact the centering ratio of the instruments used for shaping the root canals.

## Patient's or guardian consent

Not applicable as it is an in-vitro study.

## Ethical clearance

This study was approved by the Institutional Ethics committee (REF NO.: CDSRC/IES/2024/35.)

## Source of funding

None.

## Declaration of competing interest

The authors declare that they have no known competing financial interests or personal relationships that could have appeared to influence the work reported in this paper.
